# Supplementation of guanidinoacetic acid and rumen-protected methionine increased growth performance and meat quality of Tan lambs

**DOI:** 10.5713/ab.22.0008

**Published:** 2022-04-30

**Authors:** Jian Hao Zhang, Hai Hai Li, Gui Jie Zhang, Ying Hui Zhang, Bo Liu, Shuai Huang, Jessie Guyader, Rong Zhen Zhong

**Affiliations:** 1 Departments of Animal Science, School of Agriculture, Ningxia University, Yinchuan 750021, China; 2 Evonik (China) Co., Ltd., Beijing 100600, China; 3 Evonik Operations GmbH, Rodenbacher Chaussee 4, Hanau 63457, Germany; 4 Jilin Provincial Key Laboratory of Grassland Farming, Northeast Institute of Geography and Agroecology, Chinese Academy of Sciences, Changchun, Jilin 130102, China

**Keywords:** Guanidinoacetic Acid, Meat Quality, Nitrogen, Rumen Protected Amino Acid, Sheep

## Abstract

**Objective:**

Tan lambs (n = 36, 3 mo old, 19.1±0.53 kg) were used to assess effects of dietary guanidinoacetic acid (GAA) and rumen-protected methionine (RPM) on growth performance, carcass traits, meat quality, and serum parameters.

**Methods:**

Lambs were randomly assigned to three treatment groups, with 6 pens per group and 2 lambs per pen. Dietary treatments were: basal diet alone (I); basal diet supplemented with 0.08% GAA+0.06% RPM (II); and basal diet supplemented with 0.08% GAA+0.08% RPM (III). Diets were provided three times a day for 90 d. Intake per pen was recorded daily and individual lamb body weight (BW) was measured monthly. Carcass traits were measured after slaughter and meat quality at the end of the experiment, blood samples were taken on a subgroup of lambs for analysis of indicators mostly related to protein metabolism.

**Results:**

Final BW and average daily gain for the first and second month, and for the entire experiment were greater in Treatment II compared to Treatment I (p<0.05), whereas feed to gain ratio was lower (p<0.05). Treatment II had the optimal dressing percentage and net meat weight proportion, as well as crude protein and intramuscular fat concentrations in muscles. Treatment II improved meat quality, as indicated by the greater water holding capacity, pH after 45 min and 48 h, and lower shear force and cooking loss. Dietary supplementation of GAA and RPM also increased the meat color a* and b* values at 24 h. Finally, Treatment II increased total protein, and serum concentrations of albumin and creatinine, but decreased serum urea nitrogen concentrations, indicating improved protein efficiency.

**Conclusion:**

In this study, 0.08% GAA+0.06% RPM supplementation improved growth performance and meat quality of Tan lambs.

## INTRODUCTION

In ruminants, supplementing guanidinoacetic acid (GAA) and methionine (Met) may improve meat-producing performance. GAA is a natural precursor of creatine, which is essential in muscle mass development through its role in ATP regeneration [1,2]. In previous studies, dietary supplementation of GAA increased average daily gain (ADG) of both lambs (supplemented 0.08% to 0.12% GAA on dry matter [DM] basis [3]) and bulls (0.06% to 0.09% DM [4]; 0.06% DM [5]) compared to controls.

It is well established that Met is one of the most limiting amino acids for growing animals, depending on dietary composition. It is involved in various key physiological processes, e.g., protein production, and is also a methyl donor having a role in transsulfuration and DNA methylation [6]. The impact of rumen-protected methionine (RPM) on daily weight gain has not been clearly established, even though beneficial effects of protected Met on feed efficiency and meat quality were reported in lambs fed from 1.5 to 6.0 g metabolizable Met/d [7] and at the diet inclusion rate of 0.06% to 0.09%, respectively [8].

Beyond productivity, consumers are increasingly concerned about food quality [9]. Whether and how GAA and RPM can interact, and impact meat quality of ruminants is still under investigation. In pigs, dietary creatine or GAA enhanced growth performance and carcass quality, e.g., pH, color, water holding capacity (WHC), shear force, and drip loss [10,11]. However, Jayaraman et al [12] did not observe an effect of GAA supplementation on pork quality, which was inconsistent with previous results. Li et al [13] reported that adding 0.2% GAA in diets improved feed efficiency and beef growth and quality. In an assessment of the impacts of GAA on carcass characteristics of ruminants, Chao et al [3] reported more meat and intramuscular fat, and lower carcass fat content in sheep with GAA supplemented up to 0.12% DM. Regarding RPM, existing results are contradictory. Liu et al [8] reported improved meat quality (higher carcass fat weight and grid reference tissue depth, and lower drip loss) of lambs fed 0.06% metabolizable Met/d respectively. El-tahawy et al [14] reported that adding 3.3 g/kg Met in diet improved performance of Rahmani Lambs, whereas no effect was observed with steers given 8 g metabolizable Met daily [15].

Before being exported to the muscles, creatine is synthesized in the liver from GAA and S-adenosylmethionine originating from Met [1]. Consequently, we hypothesized that feeding ruminants a combination of GAA and RPM may maximize conversion of GAA to creatine, increasing energy supply for the muscle, and promoting growth performance. Moreover, as the uptake of Met for GAA conversion may induce a methyl-group deficiency and a subsequent accumulation of homocysteine, adequate Met supply is required [16].

Therefore, the purpose of this experiment was to study effects of combining GAA with RPM on growth performance, carcass traits and meat quality of Tan lambs, and to provide guidance for their practical application in ruminant production. Two doses of RPM were tested to determine the best dosage of Met required to support creatine synthesis and other metabolic roles of Met. We focused on Tan lambs, as there is currently very limited information on the use of GAA and RPM in this breed.

## MATERIALS AND METHODS

### Animals and experimental design

All animal protocols were approved by the Animal Ethics Committee of Ningxia University (permit number NXUC 20200618). The Tan sheep used are a Chinese indigenous breed originating near the Ningxia semi-arid desert steppe and arid steppe of China. Thirty-six castrated Tan ram lambs (3 mo old) with an average initial body weight (BW) of 19.1 ±0.53 kg were used in a randomized block design. Animals were blocked by initial BW and randomly assigned to 18 pens so that each pen housing 2 lambs had similar BW at the beginning of the experiment. Pens were then allocated to one of three dietary treatments, resulting in 6 pens per treatment. The adaptation period lasted 7 d, and experimental period lasted 90 d. Lambs had free access to water throughout the experiment.

### Diet preparation and feeding

The basal diet (Table 1) was formulated according to NRC [17]. We designed the experimental treatments by referring to Chao et al [3] (production performance such as final BW and ADG were maximized when GAA was supplemented at a dose of 0.08%) and Liu et al [8] (the best production performance was achieved when RPM was supplemented at a dose of 0.06%). In addition, assuming that the demand for Met increases when GAA is used, another dose of RPM (0.08%) was tested in combination with 0.08% GAA. The three diets were fed *ad libitum* three times a day (07:30, 13:00, 18:00 h, and the additives were mixed with the feed evenly before feeding: basal diet alone (Treatment I) or supplemented with (% DM) 0.08% GAA+0.06% RPM (Treatment II), or 0.08% GAA+0.08% RPM (Treatment III). A commercial rumen-protected source of dl-Met (85%) that resists ruminal degradation through an ethyl-cellulose film coating (Mepron®) was obtained from Evonik Degussa (China) Investment Co. Ltd. (Beijing, China). Its rumen bypass and intestinal digestibility coefficient were estimated at 80% [18] and 90% [19], respectively. Guanidinoacetic acid was obtained from Beijing Gendone Agricultural Technology Co. Ltd. (Beijing, China), with GAA content ≥98% (DM basis).

### Feed composition, body weight, and feed intake

Samples of the total mixed rations were collected monthly and dried at 55°C for 72 h before being ground to pass a 40-mesh screen (0.42 mm). The DM (method 930.15) content and concentrations of CP (method 990.03), crude fiber (method 991.43), Ca (method 978.02), and P (method 946.06) were analyzed according to AOAC [20]. Fiber concentration (neutral detergent fiber and acid detergent fiber) was measured according to Van Soest et al [21]. Metabolizable energy was calculated according to NRC [17].

Lambs were weighed before morning feeding at the beginning of the study and then monthly. Average daily gain was calculated as the difference between two consecutive weights divided by days on feed [(final BW – initial BW)/d on feed]. The amounts of fresh feed offered and refused were recorded daily for each pen to estimate average feed intake per pen. For calculating the feed to gain ratio (F/G), daily dry matter intake was divided by ADG.

## Carcass traits

A lamb from each pen was randomly selected for slaughter at the end of the experiment. Carcasses were cut into halves along the midline, and meat and bone were separated on the right-side before weighing. The left-side of the carcass was weighed for carcass weight. Then, dressing percentage (%) was calculated by dividing the carcass weight by shrunk live BW (BW×0.96).

### Muscle nutritional composition

After slaughter, ~50 g of the Longissimus dorsi muscle was sampled on one side of the back and stored at −20°C before use. A 10 g subsample was sliced and freeze-dried (FD-1B-80; Shanghai Haozhuang Instrument Co. Ltd. Shanghai, China). Muscle glycogen (85 mg from the freeze-dried sample) was determined by ultraviolet spectrophotometer colorimetry according to manufacturer instructions (A043-1-1; Jiancheng Bioengineering Limited, Nanjing, China). The protein content (1.0 g from freeze-dried sample) was determined with an automatic Kjeldahl nitrogen determination instrument (KjelFlex K360; Buchi, Flawil, Switzerland). The intramuscular fat content (0.5 g from freeze-dried sample) was determined according to the instructions of fat analyzer (Fat Extractor XT15; ANKOM Technology, NY, USA). The crude ash content (1.0 g from freeze-dried sample) was determined by burning at 550°C for at least 30 min according to Chinese National standard method (GB/T6438-2007).

### Meat quality

After slaughter, the probe of a pH meter (PHSJ-5; INESA Scientific Instrument Co. Ltd., Shanghai, China) was inserted in the middle cross section of Longissimus dorsi to measure pH at 45 min, 24 h and 48 h postmortem. Another set of samples of Longissimus dorsi (3×2×2 cm) were collected 24 h postmortem and kept at 4°C pending further analyses. Drip loss and cooking loss were determined according to Li et al [7]. The shear force was measured using a digital meat tenderness meter (C-LM3B), Northeast Agricultural University, Harbin, China [7]. Water holding capacity was measured with a meat water-holding capacity tester (Model RH-1000; Guangzhou Runhu Instruments Co. Ltd. Guangzhou, China). At 0, 24, and 48 h, lightness (L*), redness (a*), and yellowness (b*) as indicators of meat color, were measured with a colorimeter (CR-400; Konica Minolta Sensing, Inc., Osaka, Japan [22]).

### Serum parameters

For analysis of biochemical indicators, 10 mL of blood was drawn by jugular vein puncture from one randomly selected lamb per pen (6 lambs/treatment), just before slaughter. After clotting, samples were centrifuged at 1,000 *g*/min for 15 min. Blood serum was separated and stored at −80°C pending analysis. Serum biochemical parameters (total protein, albumin, urea nitrogen, creatinine, glucose, total cholesterol, triglyceride, and globulin) were measured with an automatic serum biochemical analyzer (BS-180; Shenzhen Mairui Company, Shenzhen, China) after defrosting at room temperature.

### Statistical analyses

Data were analyzed using the MIXED procedure in SAS (Version 8.2, SAS Institute Inc., Cary, NC, USA). In this study, the pen was used as the experimental unit. Intake, feed to gain ratio, BW, and ADG were obtained from the average of lambs in each pen. Carcass traits, muscle nutritional composition, meat quality and serum parameters were obtained by slaughtering a lamb randomly selected from each pen. Effect of treatments on growth performances was assessed according to the following model:


$${\text{Y}}_{\text{ijk}}=\mu +{\text{D}}_{\text{i}}+{\text{M}}_{\text{k}}+{\left(\text{D}\times \text{M}\right)}_{\text{ik}}+{\text{e}}_{\text{ijk}}$$

Where variable Y_ijk_ was dependent on μ as the overall mean, main fixed effects of dietary treatment D_i_ (i = Treatment I, II, and III), measurement month M_k_ (k = 1, 2, 3) and their interaction (D×M)_ik_ and e_ijk_ as the residual error.

Effects of treatments on carcass traits, muscle composition, meat quality indicators, and serum parameters were assessed with the following model:


$${\text{Y}}_{\text{ij}}=\mu +{\text{D}}_{\text{i}}+{\text{e}}_{\text{ij}}$$

Statistical differences were tested using Duncan’s multiple range tests [23]. Statistical significance was declared at p≤ 0.05. All results are reported as least squares means with standard errors.

## RESULTS

### Growth performance and carcass traits

Treatments did not impact BW of lambs during the first 2 mo of supplementation, even though the ADG was the greatest with Treatment II during the first and second month of supplementation (p<0.05; Table 2). Final BW differed among treatments (p = 0.02), with the greatest weight achieved with Treatment II (+1.66 kg relative to Treatment I). Therefore, total weight gain of lambs throughout the study was significantly higher with that treatment. Overall, lambs on Treatment II gained 18 g/d more than lambs on Treatment I (p = 0.03). Dietary treatments had no effect on average daily feed intake of lambs throughout the experiment. However, the feed to gain ratio was the lowest with Treatment II (p = 0.02). For all significant parameters, Treatment III was statistically similar to Treatments I and II.

The dressing percentage was higher with Treatment II (+3.91 percentage points compared to Treatment I, p<0.01; Table 3). The proportion of meat in total carcass weight with Treatment II was higher than with Treatment III (p = 0.03), but not different from Treatment I. The proportion of bone in total carcass weight tended to differ among treatments (p = 0.07), with lambs fed Treatments II and III having a lower bone proportion.

### Muscle nutritional composition and meat quality

Water content of Longissimus dorsi muscle was not different among groups (Table 4). Intramuscular fat concentration was the greatest with Treatment II (+1.33 percentage points relative to Treatment I, p<0.05), though not different to Treatment III, which was similar to Treatment I. The CP concentration of muscle significantly increased with Treatments II and III (+1.00 and +0.65 percentage points compared to Treatment I, respectively). Muscle samples from lambs fed Treatment III had a higher glycogen concentration than those fed Treatment I (+0.08 percentage points; p = 0.03). Finally, ash concentration in muscle samples from lambs fed Treatment II was higher than those fed Treatment III, but not different than those fed Treatment I (p = 0.04).

Drip loss was not significantly different among treatments (Table 5). Meat samples from lambs fed Treatments I and III were statistically similar in terms of cooking loss, shear force, and WHC. Inversely, compared to Treatment I, Treatment II decreased cooking loss (–3.58 percentage points, p = 0.04) and shear force (–8.89 N, p = 0.03), and increased WHC (+2.43 percentage points, p = 0.02), without being different than Treatment III. After 45 min, the meat pH was the lowest with Treatment I and the highest with Treatment II (p = 0.03). Whereas no difference was observed among treatments at 24 h, meat pH at 48 h was again the lowest with Treatment I (p = 0.03).

**Figure 1:**
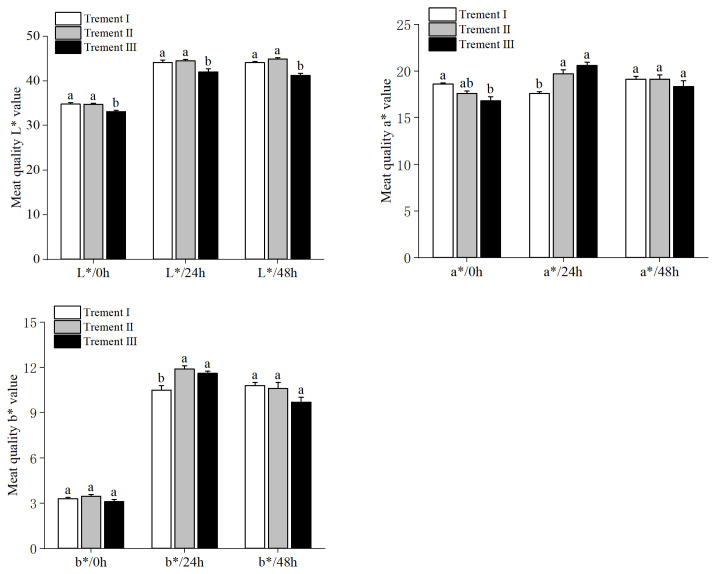
Effect of dietary GAA and RPM supplementation on meat color of lambs (L*, lightness; a*, redness; b*, yellowness). Data are presented as means±standard error of the mean. GAA, guanidinoacetic acid; RPM, rumen-protected methionine. Treatment I = basal diet alone; Treatment II = basal diet supplemented with 0.08% GAA+0.06% RPM; Treatment III = basal diet supplemented with 0.08% GAA+0.08% RPM. ^a,b^ For each parameter, bars within a sampling time without a common superscript differ (p<0.05).

Lightness at 0, 24, and 48 h was lower for Treatment III compared to the other two dietary groups (p = 0.03, Figure 1). At 0 h, redness of meat was the lowest with Treatment III (p = 0.04), whereas Treatment II was similar to both Treatments I and III. After 24 h, meat samples from Treatment I had the lowest redness value (p = 0.02), but differences were not significant after 48 h. Yellowness of meat samples was similar at 0 and 48 h, but supplemented groups (Treatments II and III) had increased yellowness of meat after 24 h (p = 0.02)

### Serum parameters

Serum concentrations in cholesterol, triglyceride, and globulin, as well as albumin/globulin ratio were similar among diets (p>0.05; Table 6). Total protein, albumin, urea nitrogen and creatinine concentrations in serum were similar between Treatments I and III, whereas Treatment II had significantly greater total protein (+0.92 g/L), albumin (+0.85 g/L), and creatinine (+4.49 μM), and significantly decreased urea nitrogen (–1.81 mM) compared to Treatment I. Serum glucose concentration was lowest in Treatment III (p = 0.01), but not significantly different than Treatment II.

## DISCUSSION

In this study, Treatment II (0.08% GAA+0.06% RPM) significantly improved growth performance of Tan lambs (higher ADG and final BW) and reduced feed conversion ratio. Using the same breed (Tan sheep) at a close initial BW (20 kg) during the same experimental duration (85 d fattening after 1-wk adaptation), Chao et al [3] also reported a higher ADG of lambs supplemented with 0.08% GAA compared to the control group (158 vs 130 g/d). This range was close to the difference in ADG observed in this study between the control and Treatment II group. This enhanced ADG was supported by variations in serum metabolites. Indeed, the serum content of protein and urea nitrogen respectively increased and decreased with this treatment. The nutritional status of the body can be understood indirectly by measuring serum total protein. Serum urea nitrogen is also an index of nitrogen balance and protein utilization; its concentration is negatively correlated with the rate of utilization amino acids for protein synthesis [24].

The beneficial effects of combining RPM with GAA (interaction) can only be assumed, as unfortunately, the experimental design did not allow us to have an additional treatment with RPM and/or GAA alone. In other words, the positive effects of Treatments II and III may also be attributed to the positive individual effects of RPM and GAA, respectively. As mentioned previously, creatine is synthesized in the liver from GAA and S-adenosylmethionine which originates from Met [1]. Ardalan et al [16] reported a methyl group deficiency after GAA supplementation in cattle without Met supplementation. Indeed, the plasma creatine concentration remained unchanged with GAA supplementation alone, whereas the plasma creatine concentration increased when GAA and RPM were supplemented simultaneously. A lack of Met can limit the conversion of GAA to creatine and limit the beneficial effects of GAA on energy metabolism and protein efficiency. In pigs, a low level of dietary Met and cysteine were a potential cause of the absence of an effect of GAA on growth [25]. Therefore, feeding GAA requires adequate levels of Met, the level of which needs to be determined. In our study, Met may have been used in the conversion of GAA to creatine, as indicated by the increased serum creatinine concentrations in lambs fed Treatments II and III. However, the net meat weight/carcass weight of Treatment III was significantly lower than that of Treatment II, which is contrary to our expectations. Majdeddin et al [27] also reported that when birds received 1.2 g/kg GAA at deficient or excessive Met levels, growth was negatively affected. This may be due to disturbances in methylation homeostasis and/or changes in Arg metabolism. High levels of GAA and RPM indicated greater methylation and s-adenosylmethionine, which may lead an increase of S-adenosylhomocysteine and inhibit most of methyltransferases. Therefore, other methylation reactions could be unbalanced in this case [27]. Moreover, when creatine is sufficient, the activity of glycine amidinotransferase in kidney will decrease, catalyzing the synthesis of GAA from Gly and Arg, thus reduced Arg metabolism to GAA.

Depending on BW, age, sex, and diet, dressing percentage for Tan lambs is usually 44% to 55% [3,7]. Our observations were with this range, with an average dressing percentage of lambs fed the basal diet alone (Treatment I) of 49.3%. This finding may be the consequence of several factors related to GAA, Met, or both. First, creatine produced from GAA can stimulate synthesis of two major contractile proteins, actin and myosin [28]. Second, dietary GAA may spare arginine normally used as a creatine precursor [29], and arginine is the most abundant nitrogen carrier of tissue protein, and it promotes skeletal muscle protein synthesis. A linear positive effect of GAA (from 0.04% to 0.12%) on dressing percentage and proportion of meat in carcass of lambs was also reported [3]. Finally, Met can increase transportation and absorption of amino acids in the small intestine and promote protein synthesis via the mechanistic target of rapamycin complex [30]. Besides, Archibeque et al [31] reported an increase in daily N retention of beef steers supplemented with RPM, even though metabolizable protein requirements were met. Interestingly, Ardalan et al [32] reported that supplementing steers with GAA improved N retention only when Met was supplemented. These studies emphasized the need for further research into the combination of GAA with methyl-donor.

Supplementing lambs with GAA and RPM also increased intramuscular fat content especially for Treatment II, and this can be again related to specific functionalities. The reverse conversion of creatine to phosphocreatine produces ATP and excess energy may be stored in intramuscular fat when phosphocreatine reaches saturation [33]. However, the intramuscular fat content of Treatment II was higher than that of Treatments III, which may be related to our previous speculation that excessive Met or excessive Met combined with GAA can inhibit creatine synthesis and thus suppress the reverse conversion of creatine to phosphocreatine and further reduce intramuscular fat deposition.

Methionine can also affect the metabolism of adipose tissue [30] and a dietary deficiency of Met may reduce the availability of lipoproteins and lipid transport. In the present work, despite the change in intramuscular fat, there were no significant differences among treatments for serum triglyceride and cholesterol concentrations.

Both Treatments II and III also increased glycogen content of muscle meat. As glycogen is the main storage form of glucose in the body, this was consistent with decreased serum glucose concentrations in lambs receiving these treatments. It was reported that creatine can stimulate muscle glycogen storage [34]. Moreover, increased Met supply may stimulate insulin secretion, and though the biological mechanisms remain unclear [35], will favor use of glucose by peripheral tissues.

It is well known that a high drip loss and a low carcass pH are associated with poor WHC which can induce liquid outflow, and loss of soluble nutrients and flavor. In addition, a decreased WHC can lead to increased L* value which is detrimental to meat color. In the present study, dietary GAA and RPM supplementation had a positive effect on the meat quality of lambs, reflected in lower cooking loss and shear force and increased WHC, pH_45 min_ and pH_48 h_, especially for Treatment II. Meat color was also improved, as indicated by the a* and b* values at 24 h and L* value, which were markedly increased by GAA and RPM. Among other causes, the higher level of protein, fat and glycogen in muscle at slaughter for lambs receiving Treatment II or III must have had a major role in improving meat quality [36,37]. Without being systematic [38], similar improvements in meat quality parameters were observed with GAA or creatine supplementation to non-ruminant species such as pigs [39] and broilers [40]. The differences among studies may result from four sources: supplements in different doses, animal species, analysis on muscle sections, or the availability of methyl-donor in the diet.

## CONCLUSION

This study highlighted the potential of dietary GAA and RPM supplementation to improve growth performance and muscle quality of lambs. When combined with 0.08% GAA, an RPM dose of 0.08% did not bring additional benefits compared to a RPM dose of 0.06% and therefore is not recommended. However further studies are necessary to refine the dosage of these two combined products in order to maximize performance and meat quality of lambs. These findings are an impetus to better understand interactions between RPM and GAA in lambs.

## Figures and Tables

**Table 1:** Ingredients and nutrient composition of diets

Item	Content
Ingredients (% DM)	
Corn silage	45.0
Corn grain	32.5
Wheat bran	4.37
Soybean meal	9.70
Sunflower meal	5.00
Flax pulp	1.00
Mineral feed	0.53
CaHPO_4_	0.20
NaCl	0.70
NaHCO_3_	0.20
Premix^1)^	0.80
Nutrients^2)^	
Metabolizable energy (MJ/kg)	8.96
Crude protein (% DM)	13.1
Crude fiber (% DM)	5.28
Neutral detergent fiber (% DM)	44.2
Acid detergent fiber (% DM)	24.8
Calcium (% DM)	0.62
Phosphorus (% DM)	0.44

DM, dry matter.

1) The premix provided the following per kg of diet: Vitamin A 100,000 IU; Vitamin D 320,000 IU; Vitamin E 60 IU; Fe 1 g; Mn 1 g; Zn 0.78 g; Cu 0.27 g; Se 0.012 g; I 0.01 g.

2) Metabolizable energy was calculated [17] and other parameters were measured.

**Table 2:** Effect of dietary GAA and RPM supplementation on growth performance of lambs

Item	Treatment^1)^			SEM	p-value

	I	II	III		
BW (kg)					
Initial	19.07	19.10	19.08	0.333	0.99
1 mo	23.52	24.04	23.67	0.317	0.80
2 mo	28.14	29.16	28.64	0.339	0.51
Final	32.84^a^	34.50^b^	33.57^ab^	0.259	0.02
Total weight gain (kg)	13.77^a^	15.40^b^	14.48^ab^	0.284	0.04
ADFI (g/d)					
0 to 1 mo	1,034	1,058	1,042	24.0	0.80
1 to 2 mo	1,238	1,260	1,282	35.9	0.51
2 to 3 mo	1,445	1,518	1,477	29.6	0.32
Whole period	1,239	1,286	1,259	42.4	0.12
ADG (g/d)					
0 to 1 mo	148^a^	165^b^	153^ab^	2.9	0.04
1 to 2 mo	154^a^	170^b^	166^ab^	4.2	0.05
2 to 3 mo	157	178	165	7.6	0.53
Whole period	153^a^	171^b^	162^ab^	3.2	0.03
F/G	8.10^b^	7.52^a^	7.82^ab^	0.18	0.02

GAA, guanidinoacetic acid; RPM, rumen-protected methionine; SEM, standard error of the mean; BW, body weight; ADFI, average daily feed intake; ADG, average daily gain; F/G, whole period feed to gain ratio (ADFI/ADG).

1) Treatment I = basal diet alone; Treatment II = basal diet supplemented with 0.08% GAA+0.06% RPM; Treatment III = basal diet supplemented with 0.08% GAA+0.08% RPM.

a,b Within a row, means without a common superscript differ (p<0.05).

**Table 3:** Effect of dietary GAA and RPM supplementation on carcass traits of lambs (%)

Item	Treatment^1)^			SEM	p-value

	I	II	III		
Dressing percentage	49.3^a^	53.2^b^	50.9^ab^	0.60	<0.01
Net meat weight/carcass weight	54.6^ab^	55.7^b^	54.1^a^	0.46	0.03
Carcass bone weight/carcass weight	26.8	25.5	25.8	0.62	0.07

GAA, guanidinoacetic acid; RPM, rumen-protected methionine; SEM, standard error of the mean.

1) Treatment I = basal diet alone; Treatment II = basal diet supplemented with 0.08% GAA+0.06% RPM; Treatment III = basal diet supplemented with 0.08% GAA+0.08% RPM.

a,b Within a row, means without a common superscript differ (p<0.05).

**Table 4:** Effect of dietary GAA and RPM supplementation on muscle nutritional composition of lambs (% fresh basis)

Item	Treatment^1)^			SEM	p-value

	I	II	III		
Moisture	72.23	72.05	72.17	0.33	0.63
Crude protein	8.56^a^	9.56^b^	9.21^b^	0.21	0.02
Intramuscular fat	6.32^a^	7.65^b^	6.86^ab^	0.32	0.03
Glycogen	0.29^a^	0.35^ab^	0.37^b^	0.02	0.03
Ash	25.21^ab^	27.83^b^	24.75^a^	0.84	0.04

GAA, guanidinoacetic acid; RPM, rumen-protected methionine; SEM, standard error of the mean.

1) Treatment I = basal diet alone; Treatment II = basal diet supplemented with 0.08% GAA+0.06% RPM; Treatment III = basal diet supplemented with 0.08% GAA+0.08% RPM.

a,b Within a row, means without a common superscript differ (p<0.05).

**Table 5:** Effect of dietary GAA and RPM supplementation on meat quality of lambs

Item	Treatment^1)^			SEM	p-value

	I	II	III		
Drip loss (%)	3.13	2.97	3.07	0.14	0.15
Cooking loss (%)	63.73^b^	60.15^a^	61.67^ab^	1.06	0.04
Shear force (N)	84.21^b^	75.23^a^	80.52^ab^	2.33	0.03
WHC (%)	39.75^a^	42.18^b^	41.41^ab^	0.68	0.02
pH_45 min_	6.04^a^	6.32^b^	6.24^ab^	0.07	0.03
pH_24 h_	5.67	5.76	5.61	0.03	0.06
pH_48 h_	5.25^a^	5.31^ab^	5.33^b^	0.02	0.03

GAA, guanidinoacetic acid; RPM, rumen-protected methionine; SEM, standard error of the mean; WHC, water holding capacity.

1) Treatment I = basal diet alone; Treatment II = basal diet supplemented with 0.08% GAA+0.06% RPM; Treatment III = basal diet supplemented with 0.08% GAA+0.08% RPM.

a,b Within a row, means without a common superscript differ (p<0.05).

**Table 6:** Effect of dietary GAA and RPM supplementation on serum parameters of lambs

Items	Treatment^1)^			SEM	p-value

	I	II	III		
Total protein (g/L)	60.56^a^	61.48^b^	61.03^ab^	0.21	0.03
Albumin (g/L)	32.75^a^	33.60^b^	33.04^ab^	0.24	0.04
Urea nitrogen (mM)	11.13^b^	9.32^a^	10.73^ab^	0.57	0.02
Creatinine (μM)	71.21^a^	75.70^b^	74.28^ab^	1.11	0.02
Glucose (mM)	5.37^b^	4.54^ab^	4.38^a^	0.19	0.01
Total cholesterol (mM)	1.94	1.75	1.83	0.07	0.06
Triglyceride (mM)	0.28	0.26	0.31	0.03	0.11
Globulin (g/L)	27.47	27.87	27.22	0.24	0.39
A/G	1.19	1.20	1.26	0.04	0.09

GAA, guanidinoacetic acid; RPM, rumen-protected methionine; SEM, standard error of the mean; A/G, albumin/globulin.

1) Treatment I = basal diet alone; Treatment II = basal diet supplemented with 0.08% GAA+0.06% RPM; Treatment III = basal diet supplemented with 0.08% GAA+0.08% RPM.

a,b Within a row, means without a common superscript differ (p<0.05).
